# Enhanced Antioxidant and Antiproliferative Activities of Apple and Korean Green Chili Pepper Extracts Cultivated with Mineral Supplementation

**DOI:** 10.3390/foods14152685

**Published:** 2025-07-30

**Authors:** Ji-Sun Lim, Mi-Hee Yu, Dong Kyu Choi, Hae Won Kim, Seung-Hwan Park, Sin-Il Sin, Jong-Sang Kim

**Affiliations:** 1 KNU LAMP Research Center, KNU Institute of Basic Sciences, Kyungpook National University, Daegu 41566, Republic of Korea; lzsunny@hanmail.net; 2 Department of Nuclear Medicine, Keimyung University Dongsan Hospital, Daegu 42601, Republic of Korea; yumihee555@gmail.com (M.-H.Y.); hwkim.nm@gmail.com (H.W.K.); 3 BK21 FOUR KNU Creative BioResearch Group, School of Life Science & Biotechnology, College of Natural Science, Kyungpook National University, Daegu 41566, Republic of Korea; dongkyu@knu.ac.kr; 4 Organic Anticancer Agriculture Institute, Gurye-gun 57607, Republic of Korea; red42co@hanmail.net (S.-H.P.); ssi2466@hanmail.net (S.-I.S.); 5 School of Food Science and Biotechnology, Kyungpook National University, Daegu 41566, Republic of Korea

**Keywords:** apple, Korean green chili pepper, antioxidant, anti-proliferative activity, Nrf2, mineral-supplemented cultivation

## Abstract

Apples and Korean green chili peppers are rich in phytochemicals and recognized for their diverse bioactive properties. Given the potential to enhance these beneficial compounds, this study investigated the effects of mineral supplementation during cultivation on the antioxidant and antiproliferative activities of extracts from both crops. Mineral-enriched cultivation significantly increased the total phenolic and flavonoid contents in both crops, which was accompanied by enhanced DPPH and ABTS radical scavenging activities. Moreover, the mineral-supplemented extracts of Korean green chili pepper activated the Nrf2 signaling pathway and upregulated downstream antioxidant enzymes, including heme oxygenase-1 (HO-1), γ-glutamylcysteine ligase (GCL), and glutathione peroxidase (GPx). Notably, the mineral-supplemented Korean green chili pepper extract significantly suppressed the proliferation of human colorectal cancer cells. These findings suggest that mineral supplementation during cultivation may improve the functional quality of apples and Korean green chili peppers, supporting their potential application in cancer prevention and complementary therapeutic strategies.

## 1. Introduction

In recent years, consumer demand for organically grown fruits and vegetables has increased, largely due to their claimed health benefits and environmental sustainability. Numerous studies have reported that organic produce often contains higher concentrations of phytochemicals, such as polyphenols, flavonoids, and carotenoids, than conventionally grown counterparts [1,2]. This enhancement is generally attributed to the activation of plant defense mechanisms under greater exposure to pests, pathogens, and environmental stressors typical in organic farming.

Although many studies report higher phytochemical levels in organic produce, this effect is not universal. For example, Soltoft et al. (2010) found no significant increase in certain health-promoting compounds in organically grown onions, carrots, and potatoes compared to conventionally grown counterparts [3]. Phytochemical accumulation in organic food crops may still be limited by soil nutrient deficiencies, particularly involving essential minerals that play key roles in plant secondary metabolism. This discrepancy may stem from deficiencies or imbalances of critical minerals such as magnesium, zinc, and selenium, which serve as cofactors in biosynthetic pathways responsible for antioxidant compound production [4,5].

Minerals are known to modulate the enzymatic activities involved in phytochemical biosynthesis [6,7]. Specifically, trace elements are crucial not only for general plant health but also for the activation of enzymes related to antioxidant production. As synthetic fertilizers are restricted in organic systems, suboptimal mineral availability may hinder the full expression of functional phytochemicals in the final produce. Several studies have reported that mineral supplementation, through either natural or controlled-environment methods, can significantly enhance the phytochemical profile and biological activity of various fruits and vegetables [5,8,9,10,11].

From a human health perspective, reactive oxygen species (ROS) are strongly implicated in the pathogenesis of numerous chronic conditions, including cancer, cardiovascular disorders, and neurodegenerative diseases [12,13,14]. As such, strategies that either suppress ROS generation or promote their detoxification are considered promising in the prevention and management of chronic diseases. Natural antioxidants, commonly found in fruits and vegetables, are known to exert protective effects by enhancing endogenous cellular defense mechanisms. In particular, the Nrf2 signaling pathway plays a central role by inducing the expression of phase II detoxifying and antioxidant enzymes, thereby enhancing the cellular capacity to scavenge ROS [15,16,17].

This study was undertaken to test the hypothesis that apples and Korean green chili peppers cultivated with mineral supplementation possess enhanced antioxidant capacity—especially via Nrf2-mediated enzyme activation—and greater antiproliferative activity, compared to those grown under standard organic farming conditions. Apple and Korean green chili pepper are representative examples of fruits and vegetables commonly consumed in Korea, so these were chosen as model samples in this study. By evaluating and comparing the bioactive properties of extracts obtained from different cultivation methods, this study aims to determine whether targeted mineral supplementation can enhance the functional quality of plant-based foods. These findings may provide valuable insights for nutrition-oriented agricultural practices and support the development of functional produce tailored to health-conscious consumers.

## 2. Materials and Methods

### 2.1. Materials

Fresh apples (Malus domestica, cultivar ‘Gamhong’) and Korean green chili peppers (Capsicum annuum, cultivar ‘Sunhangilsang’) were cultivated in experimental fields located in Pyeongchang, Gangwon Province, and Gurye, Jeonnam Province, Korea, respectively. The experiments were conducted during the spring (Korean green chili pepper) and fall (apple) seasons in 2024. Both crops were grown under certified organic conditions in accordance with the Korean Organic Standards mandated by Korean Ministry of Agriculture, Food and Rural Affairs.

Two cultivation treatments were applied:

• Standard Organic Cultivation (Control: Organic): Crops were grown using compost and certified organic soil amendments, with no additional mineral supplementation.
• Mineral-Supplemented Organic Cultivation (Treatment: DSWM): In addition to organic inputs, crops were treated with deep sea water minerals (DSWM, iCOOP Natural Dream Cooperative, Gurye, Jeollanam-do, Republic of Korea) containing 27 ppm nitrogen (N), 10.8 ppm phosphorus (P), 11.59 ppm potassium (K), 57 ppm magnesium (Mg), 9.18 ppm sodium (Na), 0.86 ppm zinc (Zn), 0.02 ppm manganese (Mn), 1.17 ppm copper (Cu), 0.63 ppm molybdenum (Mo), and 0.02 ppm selenium (Se).

For Korean green chili pepper (cv. Sunhangilsang), the mineral solution (DSWM) was diluted 2000-fold and applied via soil drenching once per week for a total of seven applications, beginning after flowering. Approximately 200 mL of diluted solution was applied per plant during each treatment to ensure uniform delivery. Peppers were harvested in June 2024 at full maturity. For apple trees (cv. Gamhong), the DSWM solution was diluted 100-fold and applied by soil drenching once a month from May to August. Each tree received approximately 2–3 L per application, depending on tree size and soil moisture. In addition, foliar applications were performed using hand sprayers to ensure full leaf wetting: once each in June and July, and twice in September. Apples were harvested at commercial maturity in September 2024. All samples were thoroughly washed with distilled water, sliced to a thickness of 1–2 mm, immediately frozen at –80 °C, and then freeze-dried using a lyophilizer (Lyoph-Pride, IlsinBioBase, Yangju, Gyeonggi-do, Republic of Korea). Unless otherwise specified, all reagents and solvents used in this study were of analytical or HPLC grade and purchased from Sigma-Aldrich (St. Louis, MO, USA).

### 2.2. Preparation of Extracts

Freeze-dried apple and Korean green chili pepper samples were ground into a fine powder using a laboratory blender and stored at –20 °C in airtight containers until extraction. To prepare ethanol extracts, 3.0 g of each powdered sample was mixed with 30 mL of 80% ethanol (*v*/*v*) and subjected to extraction for 24 h in shaking incubator (VS37S, Vision Scientific Co., Ltd., Daejeon, Republic of Korea) with 200 rpm, at room temperature. The mixtures were centrifuged at 3000× *g* for 15 min at 4 °C (VS-5000i, Vision Scientific Co., Ltd.), and the supernatants were collected and were filtered through a syringe filter (0.45 μm, Corning, NY, USA). The filtrates were concentrated under reduced pressure using speed vacuum centrifuge (Speedvac, VS-250, Vision Scientific, Co., Ltd.) at room temperature to obtain the final dried extracts.

### 2.3. Determination of Total Phenolic and Flavonoid Contents

#### 2.3.1. Total Phenolic Content (TPC)

The total phenolic content of the extracts was measured using the Folin–Ciocalteu colorimetric method, as described by Singleton and Rossi [18], with slight modifications [19]. Briefly, 25 µL of each extract (1 mg/mL in ethanol) was mixed with 75 µL deionized water and 25 µL of 50% Folin–Ciocalteu reagent in a 96-well microplate and incubated for 6 min at room temperature. Then, 100 µL of 7.5% sodium carbonate (Na_2_CO_3_) solution was added to the mixture, followed by incubation at room temperature for 90 min in the dark. Absorbance was measured at 765 nm using a microplate reader (Gen5, Agilent BioTek, Santa Clara, CA, USA). Gallic acid (Sigma-Merck, St. Louis, MO, USA) was used as a standard, and the results were expressed as milligrams of gallic acid equivalents per gram of dry extract (mg GAE/g dry weight).

#### 2.3.2. Total Flavonoid Content (TFC)

The total flavonoid content was determined using the aluminum chloride colorimetric method [20,21]. In brief, 25 µL of each extract (1 mg/mL) was mixed with 100 µL of deionized water and 10 µL of 5% sodium nitrite (NaNO_2_) in a 96-well microplate (SPL Life Sciences, Pocheon, Gyeonggi-do, Republic of Korea). After 5 min, 15 µL of 10% aluminum chloride (AlCl_3_) was added, followed by 50 µL of 1 M sodium hydroxide (NaOH) and 50 µL deionized water after an additional 6 min. The absorbance was read at 510 nm. Quercetin was used as a standard, and TFC was expressed as milligrams of quercetin equivalents per gram of dry sample (mg QE/g dry weight).

All measurements were performed in triplicate, and the results are presented as means ± standard deviation (SD).

### 2.4. Antioxidant Activity Assays

#### 2.4.1. DPPH Radical Scavenging Assay

The antioxidant activity of the extracts was assessed using the DPPH (2,2-diphenyl-1-picrylhydrazyl) radical scavenging assay, according to the method of Brand-Williams et al. [22], with slight modifications. Briefly, 100 µL of extract solution at various concentrations (25–400 µg/mL) was mixed with 900 µL of 0.1 mM DPPH solution in methanol. The mixture was vortexed and incubated in the dark at room temperature for 30 min. Absorbance was measured at 517 nm using the microplate reader (Gen5, Agilent BioTek). The radical scavenging activity was calculated using the following equation:

DPPH radical scavenging activity (%) = [1 − (A_control_ − A_sample_)] × 100, where A is the absorbance of the DPPH solution without extract. Quercetin was used as a positive control.

Antioxidant activity was expressed as the IC_50_ value (mg dry sample/mL), defined as the extract concentration required to scavenge 50% of the DPPH radicals.

#### 2.4.2. ABTS Radical Cation Scavenging Assay

ABTS [2,2′-azinobis-(3-ethylbenzothiazoline-6-sulfonic acid)] radical scavenging activity was measured based on the method of Re et al. [23], with modifications. ABTS radical cation (ABTS^•+^) was generated by mixing 7 mM ABTS stock solution with 2.45 mM potassium persulfate and allowing the mixture to react in the dark at room temperature for 12–16 h. The ABTS^•+^ solution was then diluted with ethanol to obtain an absorbance of 0.70 ± 0.02 at 734 nm.

To perform the assay, 100 µL of extract (at various concentrations) was added to 900 µL of ABTS^•+^ solution, and the mixture was incubated at room temperature for 6 min. The absorbance was measured at 734 nm, and the scavenging activity was calculated using the same formula as in the DPPH assay. Quercetin was used as a standard antioxidant.

Antioxidant activity was expressed as the IC_50_ value (mg dry sample/mL), defined as the extract concentration required to scavenge 50% of the ABTS radicals.

All assays were conducted in triplicate, and results are presented as means ± standard deviation (SD).

### 2.5. Nrf2-Mediated Antioxidant Enzyme Assay Using HCT116-ARE Reporter Cells

The activation of the Nrf2 signaling pathway by apple and Korean green chili pepper extracts was evaluated using a stably transfected HCT116-ARE-luciferase reporter cell line, which contains a luciferase gene under the control of an antioxidant response element (ARE) promoter.

#### 2.5.1. Cell Culture and Treatment

HCT116 cells stably transfected with an antioxidant response element (ARE)-driven luciferase reporter gene were used in this study, as previously established in our lab [24]. Cells were cultured in Dulbecco’s modified Eagle medium (DMEM; Welgene Inc., Gyeongsan, Gyeongsangbuk-do, Republic of Korea) supplemented with 10% fetal bovine serum (FBS; Welgene Inc.), 1% penicillin–streptomycin (HyClone Laboratories, part of Cytiva, Marlborough, MA, USA), 2% HEPES buffer (HyClone Laboratories), and 1% non-essential amino acids (MEM NEAA; Gibco, Thermo Fisher Scientific, Waltham, MA, USA). The cells were maintained at 37 °C in a humidified incubator (MCO-19AIC, Sanyo, Osaka, Japan) with 5% CO_2_.

When cultures reached approximately 80% confluency, cells were subcultured by washing with phosphate-buffered saline (PBS) and detaching with trypsin-EDTA solution (Welgene Inc.). For routine passaging, cells were seeded at a density of 1 × 10^5^ cells per 100-mm culture dish (SPL Life Sciences, Pocheon, Republic of Korea).

#### 2.5.2. Reporter Assay

For the luciferase assay, cells were seeded in 6-well white opaque plates at a density of 5 × 10^5^ cells/well and allowed to attach overnight [24]. The cells were then treated with different concentrations (200 μg/mL) of each extract (apple and Korean green chili pepper, control and mineral-supplemented) dissolved in DMSO (final DMSO concentration < 0.1%). After 24 h of treatment, the cells were lysed, and luciferase activity was measured using a commercial luciferase assay system (Promega, Madison, WI, USA) according to the manufacturer’s instructions. Luminescence was recorded using a microplate reader (GloMax Explorer System, Promega Corp., Madison, WI, USA). Luciferase activity was normalized to cell viability determined using the Bradford assay and expressed as fold induction relative to untreated control.

### 2.6. ROS Scavenging Activity Assay

The antioxidant effects of apple and Korean green chili pepper extracts were evaluated using the 2′,7′-dichlorofuorescein diacetate (H_2_DCFDA; Sigma Aldrich, St. Louis, MO, USA), a non-fluorescent probe that, once hydrolyzed intracellularly, is oxidized by ROS to generate the fluorescent product 2′, 7′-dichlorofuorescein. Briefly, HCT116 were seeded in a black-bottom 96-well plate (Nunc, Rochester, NY, USA) at a density of 2 × 10^4^ cells/well and incubated for 24 h. Subsequently, the cells were treated with apple and Korean green chili pepper extract (100 and 200 μg/mL) for an additional 24 h. Following treatment, cells were incubated with H_2_DCFDA (20 μM) for 30 min. Fluorescence intensity was measured using a fluorescence microplate reader (Infinite 200; Tecan, Grodig, Austria) with excitation and emission wavelengths set at 485 nm and 535 nm, respectively.

### 2.7. Protein Expression Analysis of Antioxidant Enzymes

HCT116 were seeded in a 6-well plate at a density of 4 × 10^5^ cells/well and incubated for 24 h. The cells were treated with apple and Korean green chili pepper extract (100 and 200 μg/mL) for an additional 24 h. Cells were lysed with radioimmunoprecipitation assay buffer (EBA-1149, ELPIS BIOTECH, Daejeon, Republic of Korea). Samples were centrifuged at 13,000 rpm for 30 min at 4 °C. Protein concentrations were measured using the Bradford assay (Bio-Rad Laboratories, Hercules, CA, USA). Equal amounts of protein (30 μg) were loaded onto 10% SDS-PAGE gels for electrophoresis and subsequently transferred to polyvinylidene difluoride membranes. Immunoreactive bands were detected using the SuperSignal West Femto Maximum Sensitivity Substrate (34096, Thermo Fisher Scientific, Waltham, MA, USA), and band intensities were analyzed using Scion Image software and the FUSIONSOLO5 imaging system (KOREA BIOMICS, Seoul, Republic of Korea). The following primary antibodies were used: anti-heme oxygenase-1 (HO-1; ab13243, Abcam), anti-glutathione peroxidase 4 (GPx4; sc-166570, Santa Cruz Biotechnology, Dallas, TX, USA), anti-γ-glutamylcysteine synthetase (GCLC; sc-390811, Santa Cruz Biotechnology), and anti-β-actin (2118, Cell Signaling Technology, Danvers, MA, USA). Secondary antibodies included HRP-conjugated anti-mouse IgG (1:1000, Santa Cruz Biotechnology) and anti-rabbit IgG (1:1000, Santa Cruz Biotechnology).

### 2.8. Antiproliferative Activity Assay

The antiproliferative effects of apple and Korean green chili pepper extracts were evaluated using the Cell Counting Kit-8 (CCK-8; Dojindo Laboratories, Kumamoto, Japan), which is based on the reduction of water-soluble tetrazolium salt (WST-8) by cellular dehydrogenases in viable cells.

#### 2.8.1. Cell Culture and Treatment

Human colorectal carcinoma cells (HCT116) were obtained from Korean Cell Line Bank (Seoul, Republic of Korea) and cultured in DMEM (WelgeneInc.) supplemented with 10% fetal bovine serum (FBS), 1% penicillin–streptomycin, and maintained in a humidified 5% CO_2_ incubator at 37 °C.

Cells were seeded into 96-well plates at a density of 5 × 10^3^ cells per well and allowed to attach for 24 h [25]. Subsequently, the cells were treated with varying concentrations (0.25, 0.5, and 1 mg/mL) of freeze-dried apple and Korean green chili pepper extracts (from both control and mineral-supplemented cultivation) for 72 h. The final concentration of DMSO used as the extract solvent was kept below 0.1% in all wells, including controls [25].

#### 2.8.2. CCK-8 Assay

After treatment, 10 μL of CCK-8 solution was added to each well, followed by incubation for an additional 2 h at 37 °C. The absorbance was measured at 450 nm using a microplate reader (Bio Tek Synergy H1 Microplate Reader, Winooski, VT, USA) r. Cell viability was calculated as a percentage relative to untreated control cells, and antiproliferative activity was expressed as the percentage of cell growth inhibition.

### 2.9. Statistical Analysis

Statistical analyses were performed using IBM SPSS Statistics software (version 25.0; IBM Corp., Armonk, NY, USA). Differences between groups were evaluated using Student’s *t*-test, and a *p*-value less than 0.05 was considered statistically significant. Significant differences were denoted using distinct alphabetical letters, asterisks (*), or hashtags (#), as appropriate.

## 3. Results and Discussion

### 3.1. Total Phenolic and Flavonoid Contents

#### 3.1.1. Total Phenolic Contents

Apples (cv. ‘Gamhong’) and Korean green chili peppers (cv. ‘Sunhangilsang’) cultivated under different conditions were sliced, freeze-dried, ground into powder, and extracted with 80% ethanol for total phenolic content analysis. As shown in Table 1, apples grown with mineral supplementation (DSWM) exhibited the highest total phenolic content (TPC) among all tested samples. Specifically, apples cultivated under standard organic practices contained 4554 ± 407 μg/g dry weight, whereas those supplemented with DSWM yielded 5779 ± 453 μg/g dry weight—a statistically significant increase (*p* < 0.05). Reported TPC values for fresh apple range from 164 to 561 μg GAE/g fresh weight, while apple peels contain higher levels, ranging from 782 to 2012 μg GAE/g fresh weight [26]. Similarly, D’Abrosca et al. [27] reported TPC values of 760 μg/g in apple skin and 317 μg/g in the flesh [26]. Considering that our study used whole, dried apple samples, the TPC values observed are broadly consistent with those reported in the literature when accounting for water content and tissue differences.

A similar trend was observed in Korean green chili peppers: mineral-supplemented samples contained 4569 ± 283 μg GAE/g dry weight, compared to 2509 ± 283 μg GAE/g dry weight in the control group, also representing a significant enhancement (*p* < 0.05). Previous studies have reported varying TPC values for Korean green chili peppers. Shaha et al. (2013) documented TPC values ranging from 1012 to 1206 μg GAE/g fresh weight [27], while Khatun et al. (2022) reported values between 2080 and 2240 μg GAE/g dry weight [28], which are consistent with the results of the present study.

#### 3.1.2. Total Flavonoid Contents

For flavonoid analysis, the same extraction procedure was applied. As shown in Table 2, apples cultivated with DSWM exhibited the highest total flavonoid concentration. Organically grown apples contained 6847 ± 643 μg QE/g dry weight, whereas mineral-supplemented apples recorded 11,780 ± 2498 μg QE/g dry weight, representing a statistically significant increase (*p* < 0.05). Previous studies reported flavonoid levels in apple skin and pulp as 47.8 mg QE and 16.0 mg QE per 100 g fresh weight, respectively [29]. Another study found that freeze-dried apple powder contained total flavonoid contents ranging from 276 to 504 mg QE per 100 g sample, which is notably lower than the levels observed in our mineral-treated apples [30].

Similarly, Korean green chili peppers treated with DSWM showed significantly higher flavonoid content than their organically cultivated counterparts. Organically grown Korean green chili peppers contained 2430 μg/g dry weight, while mineral-supplemented Korean green chili peppers contained 4252 μg/g dry weight—approximately 1.7 times greater. Previous studies have reported variable TFC values in chili peppers depending on cultivar and plant part. Ab Rahman et al. (2024) reported 21.65 and 16.43 mg QE/g dry weight in the pericarp and seeds, respectively [31]. Another study observed TFC levels comparable to ours, with values around 3.28 mg catechin equivalents per gram of sample. Additionally, Ribes-Moya et al. (2020) reported a maximum of 287 mg/kg TFC, calculated by summing individual flavonoid contents, and found no significant differences in TFC between conventionally and organically grown peppers [32]. This suggests that flavonoid content is influenced by various factors, including genotype, environmental conditions, maturity stage, and plant part, in addition to cultivation practices such as organic or conventional farming.

Overall, our findings suggest that DSWM treatment may enhance flavonoid biosynthesis in both apple and Korean green chili pepper crops.

Interestingly, in our study, apples (cv. Gamhong) exhibited higher total flavonoid content (TFC) than total phenolic content (TPC), whereas Korean green chili peppers (cv. Sunhangilsang) demonstrated the opposite trend, with TPC values exceeding TFC. This divergence likely reflects intrinsic phytochemical differences between the two species [33,34,35]. Apples are particularly rich in flavonols—especially quercetin and its glycosides—which dominate their phenolic profile [36]. Flavonols can account for over 70% of the total polyphenols in apple peels, with common constituents including hyperoside, quercitrin, and rutin [37]. In contrast, Korean green chili peppers tend to accumulate a broader spectrum of phenolic acids—such as catechin, epicatechin, apigenin, chlorogenic acid, caffeic acid, p-coumaric acid, and ferulic acid—which frequently surpass flavonoid concentrations [38,39].

Beyond compositional differences, the discrepancy between TPC and TFC values may also result from methodological variations in the spectrophotometric assays used. The Folin–Ciocalteu agent employed for TPC determination reacts broadly with various reducing agents, including phenolic acids, with results expressed as gallic acid equivalents (GAE, MW ≈ 170.1). In contrast, the aluminum chloride colorimetric method used for TFC is more specific to flavonoid structures, with results expressed as quercetin equivalents (QE, MW ≈ 302.2). These differences in molecular weights, compound reactivity, and matrix interactions can lead to situations where TFC appears higher than TPC, even though flavonoids are a subclass of phenolic compounds. Such outcomes do not necessarily indicate analytical error but rather reflect the differential sensitivity and specificity of the two methods.

Furthermore, environmental factors such as light exposure, maturity stage, and postharvest storage can differentially influence the accumulation of flavonoids and phenolic acids across crop species. For instance, flavonol concentrations in apples may increase under high light exposure, whereas phenolic acid levels in peppers often decline as the fruit ripens [40,41]. Therefore, the observed trend—higher TFC than TPC in apples and the reverse in Korean green chili peppers—can be attributed to a combination of species-specific phytochemical composition, methodological factors, and developmental stage of the fruit. This highlights the importance of complementing spectrophotometric assays with more precise, compound-specific analytical techniques such as HPLC or LC–MS in future research.

As presented in Table 1, Korean green chili peppers grown under organic conditions showed a total phenolic content of 2509 μg GAE/g dry weight, while those cultivated with DSWM reached 4569 μg GAE/g dry weight—an approximate 1.8-fold increase. This suggests that mineral supplementation may enhance the biosynthesis or accumulation of phenolic compounds in Korean green chili peppers. However, due to the intrinsic physiological variability of pepper fruits, even under controlled cultivation, these findings should be interpreted with some caution. Differences in extraction efficiency and method specificity could also influence the quantification of phenolics and flavonoids [42]. The Folin–Ciocalteu and aluminum chloride assays differ in their chemical reactivity and sensitivity, which may yield variable results depending on the structural diversity of phenolic compounds present in each matrix. Moreover, phenolic acid content in Korean green chili peppers typically decreases during ripening, while flavonoid concentrations—especially in apples—can remain stable or increase under specific environmental stimuli such as UV or visible light exposure [43].

Thus, the observed trend—higher TFC than TPC in apples, and the reverse in Korean green chili peppers—likely reflects a complex interplay of phytochemical profiles, analytical methodology, and developmental physiology. The elevated levels of phenolic and flavonoid compounds observed in mineral-supplemented samples may, in part, explain their improved antioxidant potential. This relationship will be examined in greater detail in the subsequent section on antioxidant activity.

### 3.2. Antioxidant Activity

#### DPPH and ABTS Radical Scavenging Activity

The antioxidant capacity of 80% ethanol extracts from apple and Korean green chili pepper samples was evaluated using DPPH and ABTS radical scavenging assays. The undiluted extract of organically cultivated apples exhibited a DPPH radical scavenging activity with IC_50_ of 42.0 ± 3.3, while the extract from mineral-supplemented apples showed a significantly higher activity of IC_50_ of 38.4 ± 4.9 (Table 3). More specifically, the undiluted extract of organically cultivated apples exhibited a DPPH radical scavenging activity of 58.2%, while the extract from mineral-supplemented apples showed a significantly higher activity of 66.8% (Figure S1A). In Korean green chili peppers, DPPH radical inhibition was 45.0% in undiluted organic samples (IC_50_ = 125.7 ± 16.1) and 52.0% in mineral-supplemented samples (IC_50_ = 91.4 ± 14.6), indicating a statistically significant enhancement following mineral supplementation (Figure S1B, Table 3). For ABTS radical scavenging activity, the undiluted ethanol extracts from organic and mineral-supplemented apples showed inhibition rates of 90.9% (IC_50_ = 12.9 ± 2.0) and 92.1% (IC_50_ = 11.4 ± 1.6), respectively (Figure S2A, Table 3). Similarly, the ABTS scavenging activities in Korean green chili pepper extracts were 86.4% (IC_50_ = 3.7 ± 0.7) for organic and 90.4% (IC_50_ = 3.7 ± 2.7) for mineral-supplemented samples (Figure S2B).

Additionally, to assess the antioxidant stability upon dilution, the same extracts were tested after 8-fold dilution. As shown in Figures S1 and S2, mineral supplementation consistently enhanced radical scavenging activities in both crops. In apples (cv. Gamhong), DPPH activity was significantly higher in the mineral-supplemented group (32.6 ± 1.8%) compared to the organic group (19.4 ± 1.4%) (*p* < 0.05). A similar trend was observed in the ABTS assay, with mineral-supplemented apples showing 42.4 ± 2.6% activity versus 37.6 ± 4.2% in the organic group, though the statistical significance of this difference warrants further confirmation. In Korean green chili peppers (cv. Sunhangilsang), the DPPH radical scavenging activity increased significantly from 7.9 ± 1.1% in the organic group to 11.8 ± 1.1% in the mineral-supplemented group (*p* < 0.05). ABTS activity also increased from 52.1 ± 13.0% to 60.7 ± 19.2%, although the large variation suggests caution in interpreting statistical differences.

Overall, mineral supplementation during cultivation significantly enhanced the radical scavenging activity of both apple and Korean green chili pepper extracts, particularly in the DPPH assay. Notably, the enhancement of both DPPH and ABTS radical scavenging activities became more pronounced upon dilution, suggesting that the increased antioxidant potency is closely linked to mineral treatment.

### 3.3. Nrf2-Mediated Antioxidant Enzyme Activation

#### 3.3.1. ARE Reporter Induction Activity

To evaluate the activation of cellular antioxidant enzymes mediated by the Nrf2/ARE signaling pathway, HCT116-ARE cells—transfected with an antioxidant response element (ARE)-luciferase reporter construct—were treated with apple and Korean green chili pepper extracts, and luciferase activity was measured. Cells were seeded in 6-well plates at a density of 5 × 10^5^ cells/well and allowed to stabilize for at least 12 h before treatment. After a 24-h incubation with the extracts, the cells were harvested, and intracellular luciferase activity was quantified using a luciferase assay kit (Promega).

**Figure 1:**
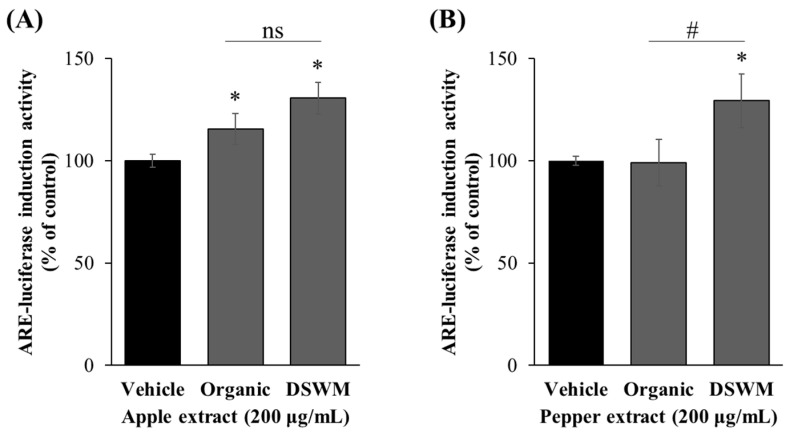
Effect of apple (**A**) and Korean green chili pepper (**B**) extracts on luciferase reporter activity in HCT116-ARE cells. Data are presented as mean ± SD from three independent experiments performed in triplicate. Asterisks and hash symbols denote statistically significant differences compared to the vehicle (*p* < 0.05, *; compared to vehicle control, #; compared to organic extract, ns = no significant difference). Organic: organically cultivated, DSWM: organically cultivated with mineral supplementation.

As shown in Figure 1A, treatment with organic and mineral-supplemented apple extract at a concentration of 200 μg/mL resulted in significantly higher luciferase activity compared to vehicle control while did not show significance between two groups. Similarly, the effects of Korean green chili pepper extracts on ARE activation were examined. As shown in Figure 1B, treatment with mineral-supplemented Korean green chili pepper extract at a concentration of 200 μg/mL resulted in significantly higher luciferase activity compared to the organically cultivated Korean green chili pepper extract at the same concentration (*p* < 0.05). These results suggest that mineral supplementation may more effectively activate the Nrf2/ARE pathway and promote the expression of antioxidant enzymes such as glutamate–cysteine ligase (GCL), and heme oxygenase-1 (HO-1), glutathione peroxidase (Gpx4), NAD(P)H:quinone oxidoreductase 1 (NQO1), glutathione reductase (GR) [44,45,46].

These findings collectively indicate that mineral supplementation during cultivation enhances the ability of both apple and Korean green chili pepper extracts to activate the Nrf2/ARE signaling pathway, potentially leading to stronger induction of cellular antioxidant defenses than organically cultivated counterparts.

#### 3.3.2. Intracellular ROS Measurement

**Figure 2:**
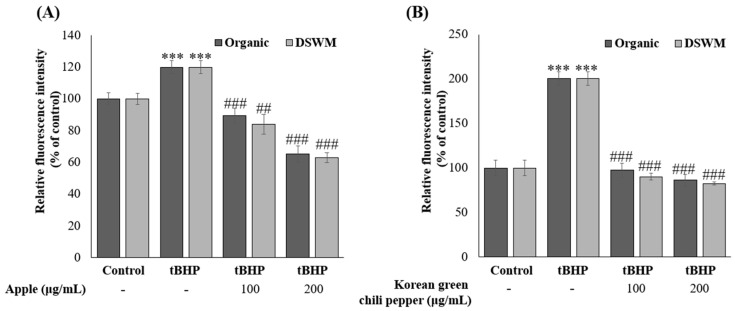
Scavenging activity of intracellular ROS levels of apple (**A**) and Korean green chili pepper (**B**) extracts in tBHP-induced oxidative stress model. Data are presented as mean ± SD from three independent experiments performed in triplicate. Asterisks and and hash symbols denote statistically significant differences compared to the vehicle (*** *p* < 0.001 vs. vehicle control, ## *p* < 0.01, ### *p* < 0.001 vs. *t*BHP-treated group). Organic: organically cultivated, DSWM: organically cultivated with mineral supplementation.

To examine whether the extracts can protect mammalian cells against oxidative stress, HCT116 cells were treated with each extract and challenged with an ROS trigger such as tBHP. The intracellular ROS levels were determined by the relative intensity of DCF (Figure 2A,B).

Treatment with the ROS inducer tert-butyl hydroperoxide (tBHP) elevated intracellular ROS levels; however, extracts from both organically cultivated and mineral-supplemented samples significantly attenuated ROS accumulation. This reduction is consistent with the elevated total phenolic content (TPC), total flavonoid content (TFC), and enhanced DPPH and ABTS radical scavenging activities observed (Table 1, Table 2 and Table 3). However, no significant difference in ROS scavenging capacity was detected between the mineral-supplemented and organically cultivated groups.

#### 3.3.3. Expression of Antioxidant Enzyme Proteins

**Figure 3:**
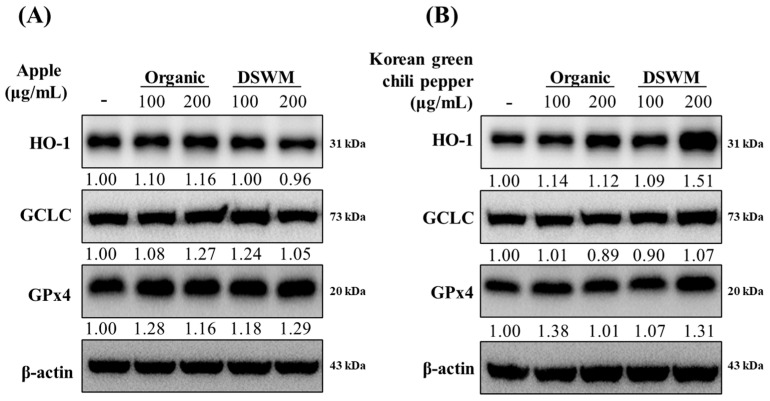
Expression levels of antioxidant enzymes in HCT116 cells treated with apple (**A**) and Korean green chili pepper (**B**) extracts. Protein expression levels were normalized to β-actin and expressed as relative intensity. - : vehicle control, Organic: organically cultivated, DSWM: organically cultivated with mineral supplementation. HO-1: heme oxygenase-1, GCLC: glutamylcysteine ligase catalytic subunit, GPx4: glutathione peroxidase 4.

To gain mechanistic insight into the antioxidant potential of apple and Korean green chili pepper extracts, we investigated the expression of key antioxidant enzymes regulated by the Nrf2 signaling pathway. As shown in Figure 3, treatment with Korean green chili pepper extracts significantly increased the expression of heme oxygenase-1 (HO-1), glutamate–cysteine ligase catalytic subunit (GCLC), and glutathione peroxidase 4 (GPx4)—all of which are critical enzymes involved in cellular defense against oxidative stress [47,48,49]. HO-1 plays a central role in the degradation of pro-oxidant heme. GCLC is the rate-limiting enzyme in glutathione biosynthesis, and GPx4 reduces lipid hydroperoxides, protecting cells from oxidative membrane damage [50,51,52]. Notably, the mineral-supplemented extract resulted in a more pronounced upregulation of HO-1 and GPx4 compared to the organic control, suggesting that mineral-enriched cultivation enhances the biosynthesis or bioavailability of Nrf2-activating phytochemicals in Korean green chili peppers.

In contrast, apple extracts—regardless of cultivation method—did not exhibit significant differences in the expression of these antioxidant enzymes, indicating a crop-specific response to mineral supplementation. This may reflect intrinsic differences in phytochemical composition or bioactivity between the two species. These findings suggest that Korean green chili pepper, when cultivated with mineral supplementation, may offer superior protection against reactive oxygen species (ROS), thereby contributing to the prevention of ROS-associated chronic diseases, including cancer, cardiovascular disease, and neurodegeneration. Overall, the enhanced activation of Nrf2-mediated antioxidant defense mechanisms by mineral-supplemented Korean green chili pepper extract underscores the potential of targeted agricultural practices in promoting functional food development for health promotion and disease prevention [53,54].

### 3.4. Antiproliferative Effects in Human Colorectal Cancer Cells

To assess the antiproliferative effects of apple extracts, human colorectal cancer HCT116 cells were treated with various concentrations of the extracts, and cell viability was evaluated. The analysis focused on comparing the effects of organically grown and mineral-supplemented apple samples in this cellular model.

HCT116 cells were seeded into 96-well plates at a density of 5 × 10^3^ cells/well and incubated for 24 h. The culture medium was then replaced with DMEM supplemented with 0.5% FBS and the respective apple extracts, followed by a 72-h incubation. After treatment, 50 μL of Cell Counting Kit-8 (CCK-8) solution was added to each well and incubated for an additional 2 h. Absorbance was measured at 450 nm using a microplate reader to assess the inhibition of cell proliferation relative to the untreated control group.

**Figure 4:**
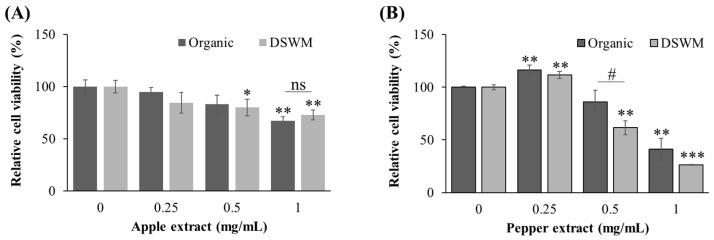
Growth inhibitory activities of apple (**A**) and Korean green chili pepper (**B**) extracts for HCT116 cells. Data are presented as mean ± SD from three independent experiments performed in triplicate. Asterisks and hash symbols denote statistically significant differences compared to the vehicle (*: *p* < 0.05, **: *p* < 0.01, ***: *p* < 0.001 vs. vehicle control; # *p* < 0.05 vs. extract at the same concentration; ns = no significant difference). Organic: organically cultivated, DSWM: organically cultivated with mineral supplementation.

In HCT116 cells, Korean green chili pepper extract from mineral-supplemented cultivation showed significant antiproliferative activity starting at a concentration of 0.5 mg/mL (Figure 4B). The IC_50_ value was calculated to be 0.9 ± 0.1 mg/mL for organic extract and 0.7 ± 0.1 mg/mL for mineral-supplemented extract. At this concentration, it induced significantly greater inhibition of cell growth than the extract from organically cultivated Korean green chili pepper. Overall, the mineral-supplemented Korean green chili pepper extract exhibited a stronger inhibitory effect on cancer cell growth than its organic counterpart. These findings suggest that cultivation practices may influence the levels or activity of bioactive compounds responsible for anti-proliferative effects.

In contrast, both organically cultivated and mineral-supplemented apple extracts demonstrated significant antiproliferative effects at 1 mg/mL (Figure 4A), but no significant difference was observed between the two cultivation methods. The IC_50_ values were estimated to be 1.5 ± 0.0 mg/mL for organic extract and 3.8 ± 2.4 mg/mL for mineral-supplemented extract. These results indicate that while apple extracts exhibit antiproliferative activity against HCT116 cells, the cytotoxic effects are modest and appear to be independent of mineral supplementation. This contrasts with the findings for Korean green chili pepper, suggesting that the enhancement of anti-proliferative activity by mineral supplementation may be crop specific.

This study has several limitations. First, the plant materials were obtained from a single growing season, which may not capture seasonal or environmental variability in phytochemical profiles. Second, all biological assays were conducted in vitro, limiting the direct extrapolation of results to in vivo systems. Third, the concentrations of extracts required to induce Nrf2-mediated antioxidant enzyme expression and inhibit cancer cell proliferation were relatively high—likely exceeding physiologically achievable levels through normal dietary intake. Fourth, the study did not identify or quantify specific bioactive compounds responsible for the observed effects, limiting mechanistic insight.

Despite these limitations, this study offers foundational evidence that mineral supplementation during cultivation can enhance the functional properties of plant-derived foods. Consistent improvements in antioxidant potential, Nrf2 pathway activation, and antiproliferative activity—particularly in Korean green chili pepper—underscore the potential of aligning agricultural practices with health-promoting food development. The use of widely consumed crops such as apples and Korean green chili peppers enhances the translational relevance of the findings. Moreover, applying a commercially available mineral supplement under organic farming conditions offers a practical and scalable approach for producing value-added crops that may appeal to both producers and health-conscious consumers.

Importantly, this research bridges agricultural inputs and biomedical outcomes by linking cultivation methods to cellular-level bioactivities, providing a strong rationale for further mechanistic and in vivo studies. These findings contribute to the growing “farm-to-function” paradigm and support the development of functional foods for chronic disease prevention.

To address current limitations, future studies should incorporate multi-seasonal sampling, in vivo validation using animal models, and detailed phytochemical profiling to identify key bioactive compounds. Ultimately, well-designed human clinical trials will be essential to confirm the applicability of these findings to dietary or therapeutic interventions.

## 4. Conclusions

This study systematically evaluated the antioxidant and antiproliferative activities of apple and Korean green chili pepper extracts cultivated with or without mineral supplementation. The findings clearly demonstrated that mineral supplementation during cultivation significantly enhanced the functional properties of both crops, as evidenced by elevated total phenolic and flavonoid contents, as well as improved radical scavenging activity measured by DPPH and ABTS assays. These enhancements suggest that mineral nutrients can promote the biosynthesis or accumulation of health-beneficial phytochemicals in plant tissues. Importantly, the biological relevance of these compositional changes was confirmed through functional assays. Mineral-supplemented Korean green chili pepper extracts not only activated the Nrf2 signaling pathway—leading to increased expression of downstream antioxidant enzymes such as HO-1, GCL, and GPx—but also exhibited greater antiproliferative effects against HCT116 colorectal cancer cells compared to their organically cultivated counterparts. This finding highlights the potential of targeted agricultural interventions to modulate cellular defense mechanisms and inhibit cancer cell growth. In contrast, while apple extracts from both cultivation methods exhibited significant antiproliferative effects, no statistically significant difference was observed, suggesting a crop-specific response to mineral supplementation. These results provide strong preliminary evidence that mineral supplementation can be strategically applied to enhance the nutraceutical value of fruits and vegetables, thereby contributing not only to consumer health but also to the development of value-added agricultural products. Nevertheless, the results should be interpreted in the context of certain limitations, including the use of in vitro models, the relatively high extract concentrations required to elicit biological effects, and the focus on a single growing season. Future research should include chemical profiling to identify the specific bioactive compounds responsible for these effects, as well as in vivo studies and human trials to validate the health benefits of mineral-enriched produce under realistic dietary conditions. Overall, this study underscores the importance of integrative “farm-to-function” strategies that link cultivation practices with human health outcomes and food innovation.

## Figures and Tables

**Table 1:** Total phenolic content in freeze-dried apple and Korean green chili pepper extracts cultivated under standard organic conditions or with mineral supplementation.

Crop	Cultivar	Cultivation Method	Total Phenolics (μg GAE/g Dry wt) ^†^
Apple	Gamhong	Organic (control)	4554 ± 407
		Mineral supplemented	5779 ± 453 *
Korean green chili pepper	Sunhangilsang	Organic (control)	2509 ± 283
		Mineral supplemented	4569 ± 283 *

^†^ Values are presented as means ± SD (n = 3). * Significantly different from the control at *p* < 0.05.

**Table 2:** Total flavonoid content in freeze-dried apple and Korean green chili pepper extracts cultivated under standard organic conditions or with mineral supplementation.

Crop	Cultivar	Cultivation Method	Total Flavonoids (μg QE/g Dry wt) ^†^
Apple	Gamhong	Organic (control)	6847 ± 643
		Mineral supplemented	11,780 ± 2498 *
Korean green chili pepper	Sunhangilsang	Organic (control)	2430 ± 135
		Mineral supplemented	4252 ± 33 *

^†^ Values are presented as means ± SD (n = 3). * Significantly different from the control at *p* < 0.05.

**Table 3:** DPPH and ABTS radical scavenging activities of freeze-dried apple and Korean green chili pepper extracts cultivated under standard organic conditions or with mineral supplementation.

Crop	Cultivation Method	DPPH Radical Scavenging Activity IC_50_ (mg/mL)	ABTS Radical Scavenging Activity IC_50_ (mg/mL)
Apple	Organic (control)	42.0 ± 3.3	12.9 ± 2.0
	Mineral supplemented	38.4 ± 4.9 *	11.4 ± 1.6
Korean green chili pepper	Organic (control)	125.7 ± 16.1	3.7 ± 0.7
	Mineral supplemented	91.4 ± 14.6 *	3.7 ± 2.7
Quercetin		0.092 ± 0.004	0.084 ± 0.003

* Significantly different from the organically grown sample (control) (*p* < 0.05). Values are expressed as means ± standard deviation (*n* = 3).

## Data Availability

The original contributions presented in the study are included in the article. Further inquiries can be directed to the corresponding author.

## Supplementary Materials

The following supporting information can be downloaded at: https://www.mdpi.com/article/10.3390/foods14152685/s1. Figure S1. DPPH radical scavenging activity of apple (A) and Korean green chili pepper (B) extracts. Values are expressed as mean ± standard deviation (n = 3). Figure S2. ABTS radical scavenging activity of apple (A) and Korean green chili pepper (B) extracts.
