# Supplementary figures and images for: Enhanced Antioxidant and Antiproliferative Activities of Apple and Korean Green Chili Pepper Extracts Cultivated with Mineral Supplementation
Source: Foods. 2025 Jul 30;14(15):2685. doi: 10.3390/foods14152685 (PMC12346781; doi:10.3390/foods14152685)

**Supplementary Figure S1**

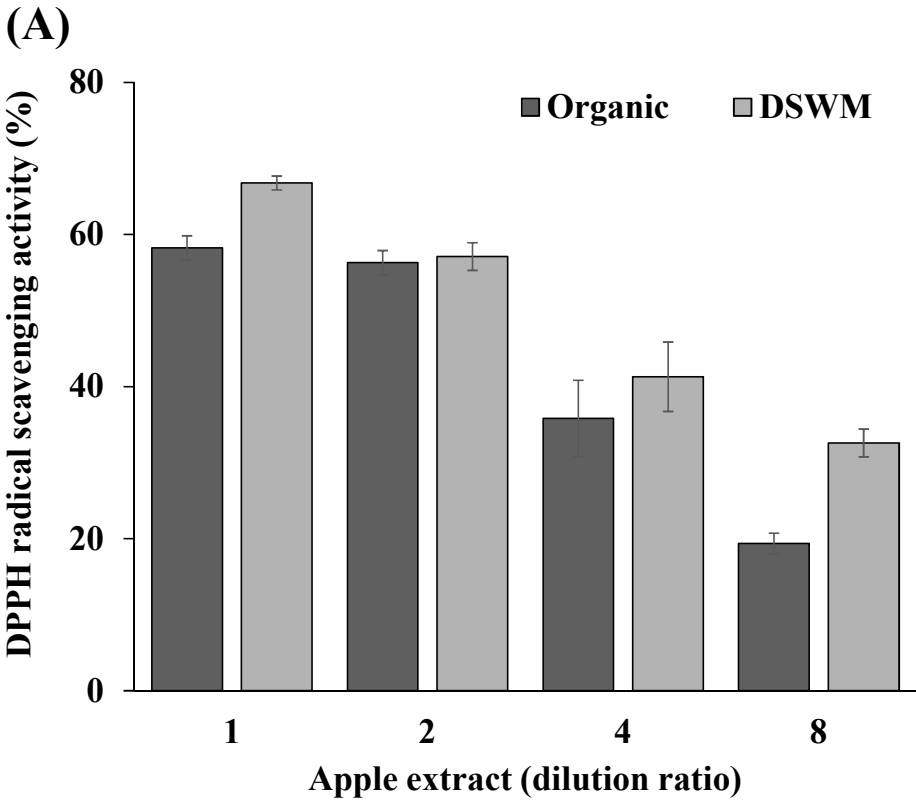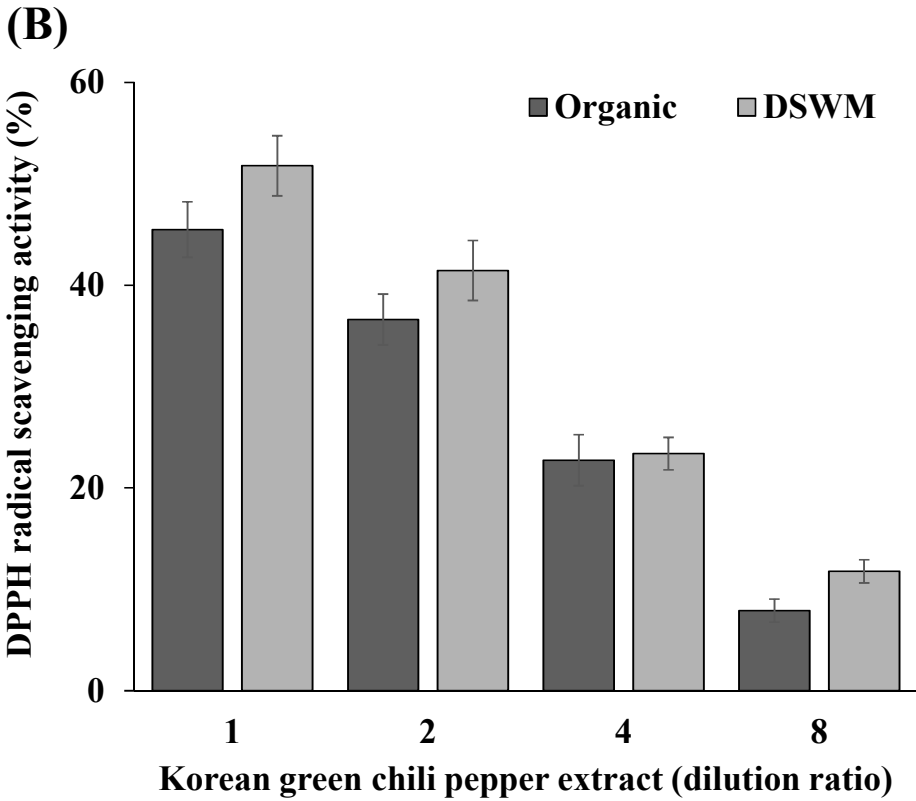

Supplementary Figure S2

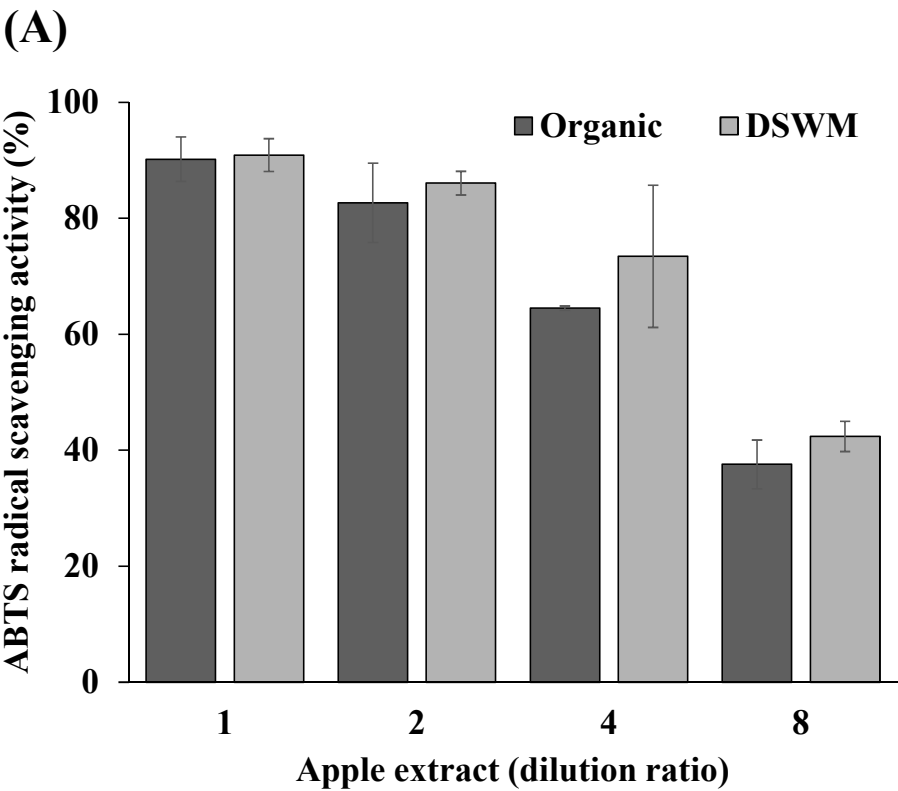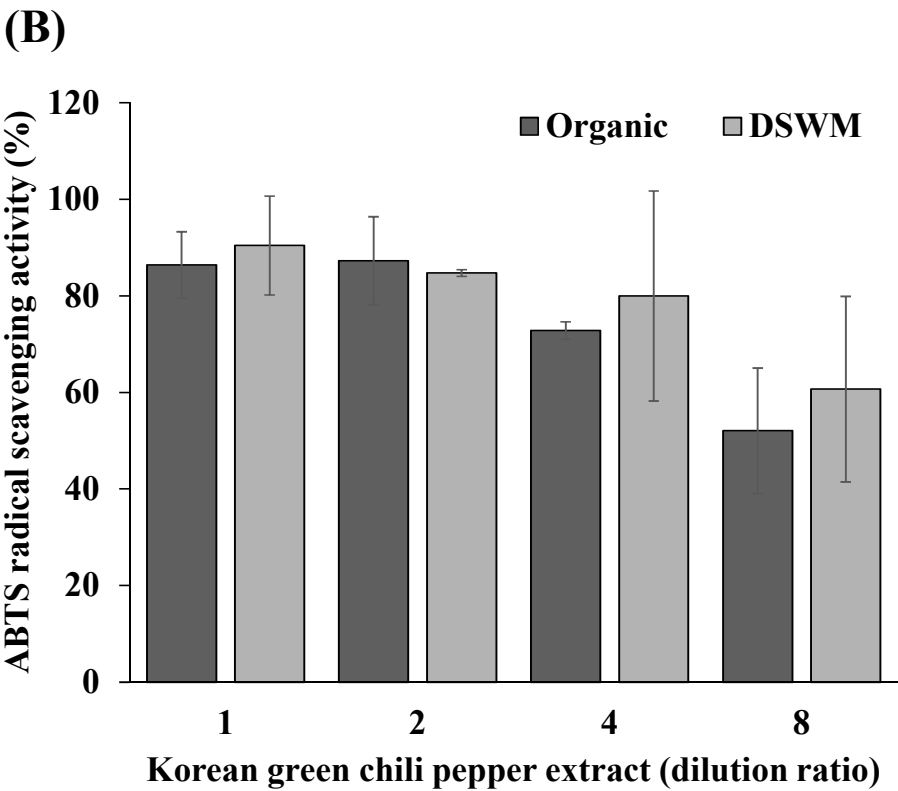

Supplement: Supplementary file 1 [file foods-14-02685-s001.zip › foods-3753331-supplementary.pdf]
